# Can a relational mindset boost analogical retrieval?

**DOI:** 10.1186/s41235-019-0198-8

**Published:** 2019-12-19

**Authors:** Micah B. Goldwater, Anja Jamrozik

**Affiliations:** 1 University of Sydney, School of Psychology, Brennan MacCallum Building (A18), Sydney, NSW 2006 Australia; 2 Independent Researcher, Place Ville Marie, Suite 400, Montreal, QC H3B 2E3 Canada

**Keywords:** Analogical retrieval, Analogy, Relational mindset, Memory retrieval

## Abstract

**Background:**

Memory retrieval is driven by similarity between a present situation and some prior experience, but not all similarity is created equal. Analogical retrieval, rooted in the similarity between two situations in their underlying structural relations, is often responsible for new insights and innovative solutions to problems. However, superficial similarity is instead more likely to drive spontaneous retrieval. How can we make analogical retrieval more likely? Inducing a relational mindset via an analogical reasoning task has previously been shown to boost subsequent relational thinking. In this paper, we examined whether inducing a relational mindset could also boost analogical retrieval.

**Results:**

We find that a relational mindset can increase analogical retrieval if induced before information is encoded in the first place, amplifying the effect of a clearly labelled relational structure. On the other hand, inducing a relational mindset at the time of retrieval did not increase analogical retrieval.

**Conclusion:**

This work further demonstrates the central importance of high-quality relational encoding for subsequent relation-based analogical retrieval, and that inducing a relational mindset can improve those encodings.

## Significance statement

Innovation and discovery in science and technology are often driven by the analogical extension of the structural relationships from one domain to another. For example, Claude Shannon’s Masters thesis extended the structure of Boolean Logic to electric circuits, which was central to the development of the computer chip. George de Mestral analysed the relationship between burs and his dog’s fur, and developed Velcro on the basis of that relationship. The Dyson vacuum is based on an analogy to the mechanism of a sawmill cyclonic separator. The list goes on.

Although the analogical extension of relational structure across domains is highly useful, there is a cognitive bottleneck holding back how readily people successfully make such extensions. The bottleneck appears to be retrieval from memory. Decades of evidence suggest that, when faced with a problem, reasoners are often not reminded of their prior knowledge that offers potential solutions based on shared structural relationships. That is, there is a failure of “analogical retrieval” and, without that retrieval, there is no source of informative relationships to apply or extend to the current problem.

The current study examined a potential tool to increase analogical retrieval— inducing a “relational mindset”. The results showed that before encoding a series of passages, inducing a relational mindset *could* increase later retrieval of those passages when faced with passages that described different semantic domains but shared relational structure. Importantly, the advantage of the relational mindset relied on the clear labelling of the initial passages’ relational structure (this labelling was unnecessary at the time of retrieval).

## Background

Innovation in design and technology, problem solving, and scientific discovery is often driven by analogical retrieval. When the current situation cues prior knowledge that shares underlying structural relationships, the retrieved relational structure can then be extended to the current situation to offer possible solutions or discoveries. For example, the bipolar plate of a fuel cell can be designed based on the structure of a leaf (see Chan et al., 2011). Although examples of real-world analogical problem solving have been well characterised and documented (e.g. Dunbar, 2000), analogical retrieval without supporting superficial similarity is relatively rare (e.g. Holyoak & Koh, 1987; Trench & Minervino, 2015). On the other hand, memories are frequently cued by situations that share superficial similarity without also sharing deeper structure (e.g. Gentner, Rattermann, & Forbus, 1993; Ross, 1989). When considering its elusiveness and utility, spontaneous analogical retrieval may be the biggest obstacle to overcome for successful problem solving (Gick & Holyoak, 1980).

Research in psychology, education, business, and design has developed methods to boost the chances of spontaneous analogical retrieval and problem solving (e.g. Catrambone & Holyoak, 1989; Gentner, Loewenstein, & Thompson, 2003; Kurtz & Loewenstein, 2007; Linsey, Markman, & Wood, 2012; Minervino, Olguín, & Trench, 2017). One of the most successful methods has been to encourage comparison of multiple analogue examples prior to problem solving. Comparing analogues highlights their common relational structure, which can both improve understanding of the relational structure in each example, and reify this common structure as a portable abstract schema (e.g. Gick & Holyoak, 1983; Goldwater & Gentner, 2015; Kurtz, Miao, & Gentner, 2001). Other manipulations can elicit similar benefits, such as framing a single learning example with an abstract schema (Mandler & Orlich, 1993), being trained to generate your own analogue problem (Bernardo, 2001) or fading out the concrete features of examples (e.g. Fyfe, McNeil, Son, & Goldstone, 2014). Like comparison of analogues, these other methods also encourage a focus on the abstract relational structure of examples, rather than superficial information. These robust, coherent, and abstract relational representations are then more likely to be cued by relevant future examples sharing relational structure and retrieved when useful (see Chen, Mo, & Honomichl, 2004 for analogical problem solving after long delays).

Encouraging comparison has been helpful both when the analogues share the key principle to solve the target problem (e.g. Gentner et al., 2003) and in open-ended problem solving when there exists no pre-established normative solution (e.g. Chan et al., 2011). However, in real-world problem-solving situations, there is no one to provide you with a useful analogue or abstract schema (Loewenstein, 2010). Furthermore, it is too late to go back and improve the quality of how you encoded the relational structure of your prior experiences. At the time of problem solving, is there anything to be done to increase the chances of retrieving the most useful prior knowledge?

One promising method comes from research suggesting it is possible to induce a relational mindset (Brown & Kane, 1988; Vendetti, Wu, & Holyoak, 2014; although see Minervino et al., 2017; Trench, Tavernini, & Goldstone, 2017; and our discussion below for different approaches). When analogical reasoning is elicited by particular task constraints or instructions, reasoners may continue to think relationally even after those task constraints or instructions are removed (hence, a relational mindset). Perhaps the earliest demonstration of a relational mindset was by Brown and Kane (1988) who showed that preschool-aged children given instruction on how to apply a solution from one problem to another would then spontaneously use an analogical strategy in further problems without similar instruction. Goldwater and Markman (2011) showed that people often use salient associations rather than relational commonalities when building categories. For example, with minimal instructional guidance on how to make categorization judgements, people were more likely to categorize a bodyguard with a celebrity (the two are associated) than a bodyguard with a force field (the two both play the same relational role—they protect others). However, when the task instructions encouraged people to compare all three before categorizing, the relational commonality between force fields and bodyguards then became the basis of categorization. Importantly for the current work, in a second set of categorization judgments, after comparison was no longer explicitly encouraged, people would continue to use relational commonalities when presented with new triplets (such as categorizing a vacuum cleaner with soap instead of vacuum cleaner with a carpet). Demonstrating that a relational mindset extends across modalities (from linguistic to visual), Vendetti et al. (2014) showed that after participants solved fill-in-the-blank analogy problems (e.g. blindness : sight :: poverty : ___), they were more likely to indicate that objects corresponded across visual scenes when they played the same role (e.g. a woman and a squirrel each receiving food) instead of sharing visual features (e.g. two women, even though one woman was giving food and the other was receiving food; task originally designed by Markman & Gentner, 1993; we will refer to this as “the picture-mapping task”).

There was a further intriguing result in that there was a correlation between fluid relational thinking ability (as measured by Raven’s Progressive Matrices (RPM); Raven, 2003) and the tendency to select the relational matches in the picture-mapping task for participants in the control condition, but there was no correlation between relational ability and relational tendency in the relational mindset condition. This pattern suggests that a relational mindset can support a focus on relational commonalities across a wide range of fluid abilities.

All of these prior results demonstrate that inducing a relational mindset increases recognition of relational commonalities among simultaneously presented stimuli. Unfortunately, as discussed above, the most difficult barrier to problem solving by analogy is not the failure to recognize the relational commonalities between multiple presented cases, but the failure of a single problem to cue an analogical match from prior knowledge (Gick & Holyoak, 1980). It is an open question whether a relational mindset might also aid relational retrieval. Why might we expect this to work, or why might we not?

A common explanation for the rarity of analogical retrieval is rooted in computational models of analogical reasoning (such as Falkenhainer, Forbus, & Gentner, 1989; Hummel & Holyoak, 1997). Critical to their explanation is that these models suggest that analogical reasoning processes are computationally intensive. Analogical reasoning requires aligning elements of two mental representations based on the common relations among them. This alignment process operates over structured mental representations, meaning that representational elements are bound together by how they relate to each other. There are three primary kinds of representational elements: entities, their attributes, and relations. Simulating this process is complex. Conceptual representations comprised of a large number of representational elements can be put into correspondence with each other in a vastly larger number of ways than smaller representations, so there are constraints on the sorts of correspondences that are preferred in this process.

Here, we will just consider a single model for brevity, the Structure Mapping Engine (SME; Falkenhainer et al., 1989; Forbus, Ferguson, Lovett, & Gentner, 2017). In the first phase of the alignment process, SME considers all possible matches between elements of the two representations (such as those based on shared attributes). Then, in a second phase to enforce structural consistency, there are two constraints. The first is “one-to-one mapping”, which ensures that an element in one representation only corresponds to one element in the other representation. The second constraint is “parallel connectivity”, which ensures that entities playing the same relational role in each representation are put into correspondence (e.g. if “Steve kissed Bill” was aligned with “Beth kissed Sally”, then Steve–Beth and Bill–Sally would be put into correspondence, and not Steve–Sally and Bill–Beth). Then a third phase imposes a third constraint, “systematicity”, that ensures more global levels of correspondence. Matches between two narratives that share higher-order themes and structure (e.g. between *Star Wars* and *Lord of The Rings*, because both depict the classic “hero’s journey” narrative) are preferred to matches where there are only superficial semantic similarities and shared lower-order relational correspondences (e.g. between *Star Wars* and *2001*, because both involve space travel).

Simulating the process of aligning relational representations is computationally expensive, and empirical evidence from humans shows that deliberately aligning relational representations is working memory intensive (e.g. Waltz, Lau, Grewal, & Holyoak, 2000), largely because the process of binding entities into relations is arguably the primary constraint on working memory capacity (e.g. Chuderski, 2014; Halford, Wilson, & Phillips, 1998). Although this relational alignment is complex, the process is feasible when only considering a pair of active representations, and people tend to succeed. However, when considering a single example (e.g. a problem or passage of text) with the aim to draw upon prior experience to assist in reasoning or comprehension, there is a lot more than just one other representation to consider.

How could memory search discover only what is relevant to the current example? It is unfeasible to fully structurally align the representation of the current example with *all prior experiences* in memory. Thus, models of analogical reasoning and retrieval (see Forbus, Gentner, & Law, 1995; Hummel & Holyoak, 1997) are hybrids in that they can both simulate full structural alignment between pairs of representations, and the process by which a current representation can cue prior experiences in memory via more simple calculation of content overlap (e.g. by calculating how many representational elements are shared between representations in Forbus et al., 1995), ignoring distinctions between deep structural and more superficial commonalities. This content overlap calculation enables the rapid comparison of a cue stimulus with vast amounts of prior knowledge.

Memory search in these models initially only considers content overlap, not full structural correspondence. This explains the rarity of analogical retrieval because structurally similar examples that share few entities and attributes have little in common overall, and so are unlikely to be retrieved based on overlapping content. On the other hand, if a present example is highly similar in its entities and attributes with a prior experience, it is quite likely that prior experience will be cued in memory. Of course, prior experiences with commonalities in relations, entities, and attributes are the most likely to be retrieved (Holyoak & Koh, 1987).

If memory retrieval is based on the degree of content overlap, and does not entail analysing structural correspondence, then how could rates of analogical retrieval increase? According to this account, the best route to increasing analogical retrieval is by increasing the proportion of what people encode from example narratives, problems, and cases as abstract relational content likely to be shared with other structurally similar cases. In simpler terms, this means improving relational understanding. This involves recognising how abstract relational principles cohere narratives, problems, and cases. A rich understanding and repertoire of abstract, coherent, relational concepts should lead to uniform encoding across the examples to which these relational concepts apply. In fact, it is the support for uniform relational encoding that Gentner and colleagues propose as the reason why case comparison increases analogical retrieval (Gentner et al., 2003; Gentner, Loewenstein, Thompson, & Forbus, 2009).

How then, according to this account, could a relational mindset improve analogical retrieval? This account suggests that a relational mindset would be most able to help analogical retrieval by encouraging a greater focus on the relational structure of example cases when they are being encoded. This greater focus would support a more uniform relational encoding across examples that share relational structure, with predicted effects similar to those of case comparison.

Most of the research on uniform relational encoding has focussed on improving the representation of initial cases encoded before a retrieval or transfer task. However, there is also evidence that the representation of examples at the time of retrieval does matter. Gentner et al. (2009) showed that comparison of two cases could help retrieval of prior analogues. This benefit is called “the late abstraction principle”. By creating a more abstract relational representation of the cue cases, these cases can serve as better cues to prior examples with matching relational structure. Trench et al. (2017) advanced evidence for the late abstraction principle by training learners to generate schematic perceptual representations of target problems, fading away concrete features. Likewise, Minervino et al. (2017) trained leaners to generate analogue problems to their current target problem. Similar to case comparison, both of these methods increased analogical retrieval and transfer when most needed, during active problem solving. To date, these two methods (of generating schematic representations and analogue problems) may represent the best chance for people to boost analogical retrieval at the time of problem solving without a helper to provide an analogue case or abstract schema.

Given existing evidence, the “uniform relational encoding” account would suggest that the most effective time for a relational mindset to have an effect would be before initial cases were encoded. However, it is still possible that a relational mindset might encourage a more relationally focussed interpretation of a cue case and improve analogical retrieval by cuing prior cases. If memory search is driven by pure content overlap between cue and prior cases, and not a more sophisticated analysis of relational structure, then the only way to increase analogical retrieval is by changing the representation of prior and cue cases.

On other hand, there is research suggesting that memory search is not solely driven by calculation of content overlap, and that analogical retrieval is more sensitive to relational structure than the above account suggests. For example, Dunbar and Blanchette (2001) have argued that the rarity of analogical retrieval in many prior studies is an artefact of task design, and that the right kind of task or prompt can engage analogical retrieval of prior knowledge to a much greater degree. An implication of this account is that people are capable of strategically retrieving different kinds of information for different purposes. If people can strategically increase analogical retrieval, and analogical retrieval is not actually that rare in the right kinds of task environments, then there is no need for a computational explanation of the rarity of analogical retrieval positing that memory search does not involve an analysis of relational structure.

This strategic retrieval account offers additional ways that a relational mindset might increase analogical retrieval. Perhaps inducing a relational mindset at the time of retrieval could boost analogical retrieval without relying on changing the representation of any cases—cue or prior. If so, this could add to the evidence discussed by Dunbar and Blanchette (2001), and challenge the computational explanation of analogical retrieval described above. Furthermore, if inducing a relational mindset could encourage the strategic retrieval of useful prior analogies, this would suggest easy and practical exercises to engage in before problem solving.

### The current research

The current experiments examined whether a relational mindset affects analogical retrieval using two sets of short passages designed by Jamrozik (2014). Pairs of passages (one in each set) expressed a specific relational concept, for example the *pre-emption* of something of lesser status or priority by something of higher status or priority. These concepts were selected because they are relevant to multiple domains of expertise, and thus relevant to educational or problem-solving settings (see Goldwater & Schalk, 2016, for a lengthy discussion). Each passage of the pair expressed the concept in a different domain, such as in law (how a federal law may pre-empt a local law when there are inconsistencies between the two) or in computer science (how a computer’s operating system may pre-empt an inessential series of computations to run a more critical program). In addition to pairs of passages sharing a relational concept from different domains, each passage shared a domain with another passage with a different relational concept. For example, the passage above on legal pre-emption shared a domain with a passage on legal proportionality—the severity of punishment should be in proportion to the severity of the crime. Across both sets of passages, every critical or “test” passage had a different relational and domain match in the other set. This allowed participants to initially read one set of passages and later read the second and report what earlier passage came to mind. Here, improved analogical retrieval was operationalized as recalling more relational matches.

We note here that we motivated this work by discussing the role of analogical retrieval and transfer in creative problem solving, and yet our task does not require any problems to be solved or new ideas to be generated. Instead, we just ask participants to say what they remember. We make this experimental choice for a couple of related reasons. First, prior research shows reminding and problem solving are intertwined processes (e.g. see Brian Ross’ research throughout the 80s and 90s: Ross, 1984, 1987, 1989; Ross & Kennedy, 1990; Ross & Bradshaw, 1994). In Mandler and Orlich (1993), all participants who explicitly recalled a prior analogue problem used that analogue to solve a current problem. In Bernardo (2001), the same experimental manipulations that boosted analogical retrieval boosted analogical problem solving.

There are obviously further important cognitive processes needed to assess the relevance of retrieved prior knowledge, and to formulate how to apply that knowledge to the present case. By focusing on retrieval, participants in the current study can report multiple cases they are reminded of without the additional demands to then draw out different solutions for each of them. It is quite possible that, with extra demands, they would only choose the first solution that came to mind. On the other hand, perhaps the pragmatics of retrieval tasks reduces analogical retrievals in comparison to problem solving tasks (see Dunbar & Blanchette, 2001). In that case, then, any evidence that a relational mindset could increase analogical retrieval could be valuable in showing that it works even when at odds with the task’s pragmatics.

Across two experiments, we examined whether and how a relational mindset may improve analogical retrieval by varying when the mindset was induced—either before encoding the first set of passages or after encoding the passages but before retrieval. Inducing a relational mindset before encoding the initial set of passages may increase analogical retrieval because of an increased focus on the relational structure in the passages, encouraging a representation in memory with robust and coherent relational structures. This novel effect would go beyond prior research showing how a relational mindset can increase a focus on the relational commonalities between co-presented examples, and offer a learning tool to make knowledge more accessible in relevant future situations.

Inducing a relational mindset after the initial encoding of passages might also improve relational retrieval. This pattern would suggest that an example with a given quality of relational encoding could be differentially accessed later. There would be two possible explanations for that effect. The first would be consistent with the late abstraction principle (Gentner et al., 2009), suggesting that changing the representation of cue cases is sufficient to help retrieval of prior analogues. The second possibility is that an induced relational mindset changes the memory search strategy of people, independent of (or in addition to) changing their representation of the cue case. If this manipulation succeeds, further research would be needed to tease these two explanations apart. Either way, these results would encourage work on how a relational mindset might improve problem solving when most needed—at the time of problem solving.

To maximise the chance of a relational mindset increasing relational retrieval after encoding, in experiment 1 all of the first set of passages included an explicit label for the relational concept they describe (such as *pre-emption* in the passage about pre-emption). Jamrozik (2014) showed that the use of a relational label at encoding increased the chances that the passage was cued by its relational match in the second set, even when the label was not present in the second passage. Jamrozik inferred that the relational label helped make the relational structure of the encoded example more prominent and coherent in memory, making it more likely to be directly cued by a later example sharing relational structure. We expected to replicate this finding (across experiments) and examined whether inducing a relational mindset would amplify this effect on the likelihood of analogical retrieval (while replicating Vendetti et al., 2014). Even with high-quality relational representations, past research shows below ceiling analogical retrieval, so an induced relational mindset could still be quite beneficial.

## Experiment 1

In the first experiment, we tested whether inducing a relational mindset after encoding of initial examples could increase analogical retrieval. The experimental sequence was as follows. First, participants read one set of passages from Jamrozik (2014), all with relational labels. Second, half of the participants completed the fill-in-blank analogy task from Vendetti et al. (2014) (taken from Green, Kraemer, Fugelsang, Gray, & Dunbar, 2009) that previously successfully induced a relational mindset. The other half of the participants completed a fill-in-the-blank semantic word association task that had very similar basic demands to the analogy task wherein three words were presented, and a fourth needed to be indicated. Unlike in the analogy task, where two domains shared the same relations, (e.g. blindness : sight :: poverty : ___; the answer being “money”) the word association task presented words all from the same domain, and seeded the fourth associated word to constrain the participants’ answers (e.g. blindness, sight, e _ _ _, glasses; the answer being “eyes”).

Next, all participants read the second set of passages (without their relational labels) and, for each passage, noted of which passages from the first set (if any) the current passage reminded them. Then, all participants completed the picture-mapping task (Markman & Gentner, 1993) which served as the primary measure of a relational mindset by Vendetti et al. (2014). In this task, participants indicated which visually depicted objects corresponded across scenes. Objects corresponded either based on common features or relations. Here, we attempted to replicate Vendetti et al.’s (2014) finding with their task. Next, all participants completed a “text-mapping” task. In this task, a subset of the test passages from encoding and retrieval was presented with their domain and relational matches, and participants rated how well they matched each (respectively). This allowed us to examine whether a relational mindset would increase recognition of common relations across passages when directly presented, even if a relational mindset did not increase retrieval. Finally, all participants completed an abbreviated version of RPM (Raven, 2003) to attempt to replicate the pattern from Vendetti et al. (2014) discussed above wherein inducing a relational mindset overcame differences in fluid thinking ability to promote a focus on relational commonalities, and potentially extend this pattern to retrieval.

In summary, this experiment presented a series of tasks identical for all participants, except for the second task, which either induced a relational mindset or did not, manipulated between participants. The basic sequence was: 1) encoding set of passages (with relational labels); 2) analogical or semantic control task; 3) retrieval set of passages (without relational labels); 4) picture-mapping task; 5) text-mapping task; and 6) RPM.

### Methods

This study was conducted with approval by the University of Sydney Human Research Ethics Committee, protocol #2013/1077, titled “Learning from Examples.”

#### Participants

Sixty undergraduate students from the University of Sydney participated in exchange for partial course credit. Participants were randomly allocated to the priming (*n* = 32) or control (*n* = 28) condition. One participant was lost at outset due to a computer error. An exclusory criterion was applied to account for participants failing to make a reasonable attempt at retrieval. Consistent with Jamrozik (2014), participants who provided no response for over half the retrieval test questions were removed from analysis. Enforcing the criterion led to the removal of another seven participants, leaving 52 (29 mindset; 23 control) in the final analysis.^1^

#### Materials and procedure

Participants were randomly assigned to either the relational mindset or control condition, and were presented with encoding passages, which were one of two sets of passages (A or B; the other set were shown at the time of retrieval to be memory cues for the encoding passages, counter-balanced across participants).

For encoding/retrieval passages, passage materials were adapted from Jamrozik and Gentner (2019) and consisted of two sets of 14 passages, typically four or five sentences long (see Additional file 1). For each participant, the encoding passages were prefaced with a sentence identifying its relational principle (e.g. “This is an example of *pre-emption*”), while the retrieval set had no such labelling preface. Each set consisted of ten test passages and four filler passages. Each test passage dealt with a particular relational concept (e.g. *trade-off*) explored in a particular domain (e.g. *medicine*). Across sets, each passage matched one passage from the other set in relational principle, and a different passage from the other set in domain. For example, in set A, the passage denoting the relational principle *trade-off* came from the domain of *medicine*. In B, the *trade-off* principle was instead explored in the *computer science* domain, and the *medicine* passage instantiated a different relational principle (*inoculation*). The four filler passages in each set also dealt with a particular relational principle set in a particular domain; however, across sets each filler passage matched only one other filler passage (both in relational principle and domain; i.e. they were literally similar).

Passages were presented on a single page, under instructions indicating participants would need to draw on the information later in the experiment, and to read the passages carefully so as to remember them. No time limit was enforced, and passage presentation order was randomized.

Following encoding, participants in the relational mindset condition were given the fill-in-the-blank analogy task. Materials for the analogy-generation task were the same 40 distant analogies (used in Vendetti et al., 2014; originally from Green et al., 2009). They were semantically “distant” in that the two pairs of words came from different domains, such as human senses and finances. Distant analogies were presented as two relational word pairs such as “blindness : sight :: poverty : money” wherein blindness is to sight as poverty is to money. The fourth term was missing, e.g. “blindness : sight :: poverty :______”. Participants were instructed to fill in the fourth term. Generating solutions requires analogical retrieval, mapping, and evaluation from the relational pattern instantiated in the base (blindness : sight) onto the target (poverty : money). See Additional file 2 for completed analogies. Trials were presented one at a time and without feedback. Participants were advised that, for some questions, multiple correct answers were possible. It was explained that as long as an answer made analogical sense, it would be marked as correct.

Following encoding, participants in the control condition were given the fill-in-the-blank semantic word-association task**.** This task was designed using the latent semantic analysis matrix application at http://lsa.colorado.edu to select 40 highly associated sets of words (e.g. “blindness, sight, eyes, glasses”). Latent semantic analysis calculates semantic relatedness based on language distribution statistics in natural text (see Landauer & Dumais, 1997). Two of the words in each set were adapted from one pair of the four-term analogies so as to maximize likeness between the tasks. One of the four terms was left incomplete (the first letter, and number of missing letters were provided), producing a hangman-style task of completing the missing word based on semantic likeness, e.g. “blindness, sight, e _ _ _, glasses” with the correct response being “eyes.” Refer to Additional file 2 for completed semantically associated word sets. As with the analogy task, trials were presented serially, no feedback was given, and participants were advised of the possibility of multiple correct answers.

We note this is not the identical control task to Vendetti et al. (2014), which used a second analogy task, but used analogies with less semantic distance between the pairs of words. All four words came from the same domain, such as the senses (e.g. blindness : sight :: deafness : hearing). Although the evidence suggests this task does not engage analogical thinking to the degree the distant analogies do, because prior analogy research suggested that detecting an effect on retrieval could be more difficult than using the picture-mapping task (as discussed above) our intuition was that a semantic association task which requires no analogical thinking whatsoever would increase our chances of measuring a difference between the two conditions, while still maintaining similar task demands (in that both tasks involve filling in a fourth word that matched a set of three in some way).

All participants then proceeded to the retrieval phase. Participants received the passage set (A or B) that they did not receive at encoding. A text entry box was positioned underneath each cue passage, and prompted participants to write down any of the original passages of which they were reminded. Participants were told they could write down multiple original passages if reminded of them by a cue passage. Similarly, participants could cite an original passage multiple times if reminded of it by multiple cue passages. Questions were presented one at a time, in a randomized order, and participants were unable to change their answers once submitted.

The number of relational retrievals and the number of domain retrievals made by each participant was calculated. Every test question retrieval (i.e. not including filler questions) was coded as either relational, domain, or other match. “Other” retrievals reflected a retrieved passage that was neither related to the test passage in relational concept nor domain. Since participants could write down multiple responses to each test question, they could each retrieve a maximum of ten relational matches, and ten domain matches overall. The number of missed responses for test questions was also calculated and included instances where no attempt was made.

Following retrieval, participants were given the picture-mapping task. The materials were the ten sets of paired scenes used in Vendetti et al. (2014) (taken from Tohill & Holyoak, 2000; based on Markman & Gentner, 1993; see Additional file 3). The pairs were comprised of two images positioned one above the other. They were presented together for 10 s. Then, an object in the top scene was highlighted. Participants were told to “click on the object in the lower scene that goes with the highlighted object in the top scene”. The instruction “goes with” was deliberately left vague so as not to bias responses. In each set, the highlighted object in the top scene matched an object in the bottom scene relationally (playing the same relational role), and matched another object in the bottom scene featurally (perceptually the same object). Click coordinates were recorded, and scores calculated as the sum of relational matches made. Thus, picture-mapping scores were out of ten and higher scores indicated greater relational orientation. This task served as Vendetti et al.’s (2014) primary measure, but that study had no intervening task between the analogy task and this one, while here the retrieval task is in between (we point out this procedural distinction here to foreshadow differences in results).

Participants then engaged with the text-mapping task. This task reused a subset of the encoding and retrieval passages (but here none had the relational label preface sentence). Each question simultaneously presented a base passage and three “related” passages, vertically stacked below. One “related” passage matched the base passage in domain, another in relational concept, and the third functioned as a distractor that was neither related to the base in domain nor relational concept. A slider was positioned alongside each “related” passage, for which participants could indicate from 0 to 100 how “related” that passage was to the base. There were four text-mapping sets in total. Two questions reused a passage from set A as the base and three passages from set B as “related” passages. The other two questions reused a base passage from B and three “related” passages from A. This was designed so that participants from either counterbalancing condition had encoded and retrieved in equal proportion the base and target passages. The order in which the three “related” passages were presented underneath the base was randomized across questions. Three scores were calculated for the text-mapping task for each participant, reflecting the average of the four relational, domain, and other ratings, respectively. They were all scaled to be out of 10, to more closely match the scores on retrieval and picture mapping.

Finally, participants completed the abbreviated RPM, which used the odd numbered questions from the standard RPM (Raven, 2003). Two additional difficult questions were included (D-11, E-11) to allow for more questions below ceiling levels of performance. This totalled 20 test questions (presented serially). Each question was worth one mark, and final scores were out of 20.

### Results

In all the analyses comparing conditions we used RPM scores as a covariate to ensure that any differences between conditions could not be explained by differences in fluid reasoning abilities (we also note that there were no statistically significant differences in RPM between conditions).

**Fig. 1:**
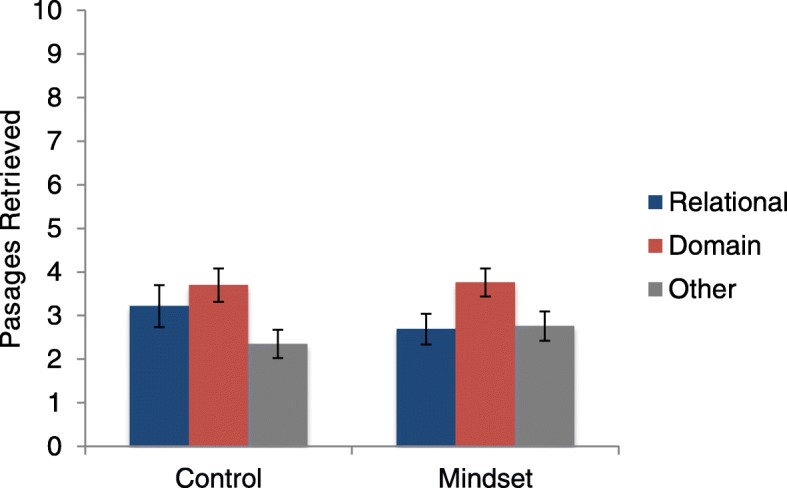
Means (and standard errors) for the number of passages retrieved in experiment 1

We conducted an analysis of covariance (ANCOVA) comparing the relational mindset condition to the control condition on relational retrievals, with RPM as a covariate. RPM showed a significant relationship with relational retrievals, *F* (1,49) = 20.97, *p* < .001, η^2^_p_ = .30. We found no evidence that the relational mindset condition (*M* = 2.69, *SD* = 1.89) increased relational retrievals. In fact, the control condition elicited more relational matches than the relational mindset condition (*M* = 3.22, *SD* = 2.30), although this difference was not statistically significant, *F* (1,49) = 2.92, *p* = .09, η^2^_p_ = .056. See Fig. 1 for the rates of the three kinds of passages retrieved. It is worth noting that, while there were still more domain retrievals than relational retrievals, these participants recalled fewer domain matches than in Jamrozik (2014); domain retrievals from E2 are higher and more similar to Jamrozik’s original findings.

**Fig. 2:**
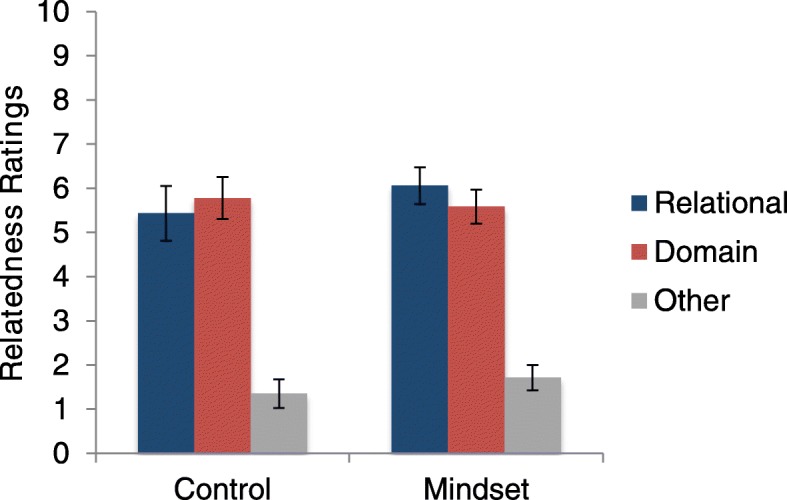
Means (and standard errors) of relatedness ratings in the text-mapping task in experiment 1. Original scale was 0–100

We conducted a second ANCOVA contrasting relational mindset and control conditions with RPM as a covariate, but now the dependent variable was the relatedness of the relational match to the base passage in the text-mapping task. Similar to the relationship between RPM and retrieval, we found a relationship between RPM and relatedness ratings, *F* (1,49) =17.65, *p* < .001, η^2^_p_ = .265. However, we found no effect of condition on relational rating (relational mindset: *M* = 6.06, *SD* = 2.22; control, *M* = 5.43, *SD* = 2.98; *F* < 1). See Fig. 2 for average ratings for all three kinds of matches. We then conducted the equivalent analysis for the picture-mapping task. Again, there was no statistically significant effect of condition (mindset: *M* = 5.79, *SD =* 2.74; control; *M* = 5.43, *SD* = 2.57; *F* < 1) but there was a significant effect for RPM as a covariate, *F* (1,49) = 11.91, *p* = .001, η^2^_p_ = .196.

Next, we analysed the relationships (correlations) among these key measures: relational retrieval, text-mapping ratings for the relational match passages, picture-mapping relational scores, and RPM scores. A key pattern in Vendetti et al.’s (2014) study was that RPM was correlated with picture-mapping scores for the control condition, but not for the relational mindset condition, suggesting that a relational mindset can overcome ability differences to encourage a relational focus. We used more tasks than Vendetti et al., (2014) (i.e. the retrieval and text-mapping task in addition to the picture-mapping task), but we hypothesized that this pattern, wherein the correlation with RPM was larger for the control than for relational mindset condition, would extend to these other tasks. To be consistent with the analysis of Vendetti et al. (2014), we examined both correlations collapsing across conditions, and also each condition independently (see Table 1).

**Table 1:** Correlations (*r* scores) between relational retrieval, ratings of the relational match passage in the text-mapping task, relational choices in the picture-mapping task, and Raven’s Progressive Matrices (RPM) scores for experiment 1

Both conditions			*N* = 52
	Retrieval	Text-mapping	Picture-mapping
Retrieval			
Text-mapping	0.72*		
Picture-mapping	0.47*	0.50*	
RPM	0.52*	0.52*	0.45*
Control condition			*n* = 23
	Retrieval	Text-mapping	Picture-mapping
Retrieval			
Text-mapping	0.75*		
Picture-mapping	0.46*	0.70*	
RPM	0.63*	0.72*	0.44*
Mindset condition			*n* = 29
	Retrieval	Text-mapping	Picture-mapping
Retrieval			
Text-mapping	0.74*		
Picture-mapping	0.51*	0.32	
RPM	0.47*	0.29	0.44*

*indicates *p* < .05.

Collapsing across conditions, all measures were significantly correlated with RPM (all *p*s < .05). The pattern was largely the same when broken down for each condition, not replicating the pattern where the correlations differed between conditions from Vendetti et al. (2014). However, we did see that differential pattern with the correlation between the relational rating from the text-mapping task and RPM. The correlation between the text-mapping relational rating and RPM in the control condition (*r* (21) = .72, *p* < .001) was significantly higher than the correlation with the mindset condition (*r* (27) = .29, *p* = .12); *z* = 2.06, *p* = .04.

### Discussion

Experiment 1 provided no evidence that inducing a relational mindset after encoding could increase the likelihood of relational retrieval. However, we also did not replicate the key pattern of results of Vendetti et al. (2014) that inducing a relational mindset would: 1) increase the relational choices in the picture-mapping task; and 2) that only the control condition would show a correlation between RPM and the picture-mapping task. Thus, it is worth asking whether we successfully induced a relational mindset.

Evidence that we did indeed induce a relational mindset came from the text-mapping task, which showed differential correlations with the picture-mapping task and RPM between the control condition (very strong relationship) and the mindset condition (not a significant relationship). There is also the possibility that we did induce a relational mindset, but then disrupted that mindset with the intervening retrieval task before the picture-mapping task could detect it. As noted above, in the study by Vendetti et al. (2014) participants performed the picture-mapping task directly after the analogy task. Inducing a relational mindset would likely have a temporary effect, and there has been no research that we are aware of examining how long the mindset lasts or what conditions or kinds of tasks maintain or disrupt it.

Given the previous success of Vendetti et al. and our methodological differences in experiment 1, we continued with the operating assumption that the analogy task does induce a relational mindset in experiment 2. To preview some results of experiment 2, we found evidence that the relational mindset does increase relational responding in the picture-mapping task (replicating Vendetti et al., 2014), but only in conditions where relational retrievals were also quite high. We speculate in the “General discussion” section that having the intervening retrieval task between inducing a relational mindset and the picture-mapping task may either maintain the mindset (if relational retrievals are frequent), or disrupt the mindset (as in experiment 1) if relational retrievals are rare.

## Experiment 2

In this next experiment, we examined whether a relational mindset could improve relational retrieval by improving the original encoding of cases. Past research showed that a relational mindset encouraged noticing more relational commonalities across pairs of scenes (Vendetti et al., 2014), or sets of words (Goldwater & Markman, 2011). However, these findings do not entail that a relational mindset will lead to a richer relational encoding of cases so that they will be more readily cued by matching relational structure after a delay. The extension to a richer relational encoding is important as it would suggest that the analogy task may be a simple exercise to engage in before a learning activity to benefit the most from it.

In this experiment, half the participants started the procedure by completing the analogy task, and the other half completed the semantic word-association task. Then every participant read an encoding set of passages. All participants then completed another word-association task to match the delay between encoding and retrieval from experiment 1. The rest of the session proceeded as in experiment 1, with the retrieval set of passages, the picture-mapping task, the text-mapping task, and finally with RPM.

Furthermore, to examine whether any benefit of a relational mindset relied on a clear relational organization in the encoding passages, we also manipulated (between participants) the use of relational language. Half of the participants got the same relational labels for the relational concepts from the first experiment (e.g. “This is an example of pre-emption”), while the passages for the other half of the participants were prefaced with a statement about the domain, for example “This is an example from computer science”, to control for the effect of labels in general. It is possible that any label could serve as a tag in the memory to help later retrieval, and so this specifically examines the effects of relational labels. Although we also note that in Jamrozik, 2014, a control condition when passages were presented without any such label (similar to the retrieval passages in the current experiments), retrieval patterns were no different than with the use of domain labels.

Experiment 2 had a 2 (mindset vs. control) × 2 (relational label vs. domain label) between-participants design.

### Methods

This study was conducted with approval by the University of Sydney Human Research Ethics Committee, protocol #2013/1077, titled “Learning from Examples.”

#### Participants

Eighty-four undergraduate students from the University of Sydney participated in exchange for partial course credit.^2^ Two participants were removed for failing to provide answers for at least half of the retrieval passages (as for experiment 1). This left 27 participants in the mindset/relational condition, 18 in the control/relational condition, 19 in the mindset/domain condition, and 18 in the control/domain condition.

#### Materials and procedure

The materials and procedure were largely the same as in experiment 1. The primary exception was that either the analogy task (in the two mindset conditions) or the semantic word-association task (in the two control conditions) were the first tasks completed, rather the reading the encoding passages first. After completing one of these two tasks, all participants read the encoding passages. Half read them prefaced with relational labels (e.g. “This is an example of *pre-emption*”), and the other half with domain labels (e.g. “This is an example from *computer science*”). The procedure was then identical for all four conditions. Participants completed an additional semantic word-association task (different items than the first set completed by the control conditions) to have a comparable delay between study and test as in experiment 1. This was followed by the retrieval passages (without domain or relational labels), the picture-mapping task, the text-mapping task, and RPM.

### Results

**Fig. 3:**
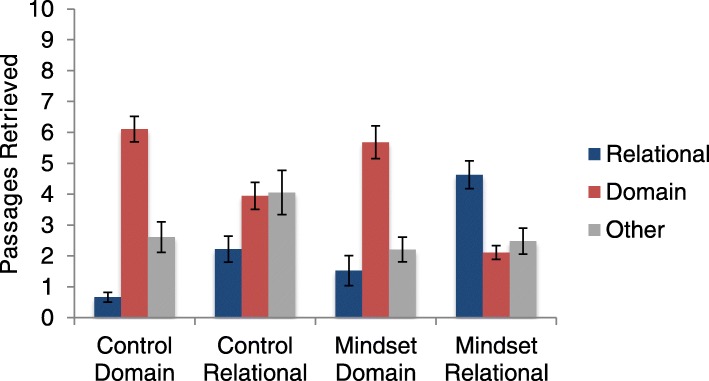
Means (and standard errors) for the number of passages retrieved in experiment 2

In all analyses comparing conditions, we used RPM scores as a covariate to ensure that any differences between conditions could not be explained by differences in fluid reasoning abilities (though here we also note that the relational language and mindset condition had the lowest RPM scores, and highest rates of relational retrieval). First, we conducted a 2 (mindset vs. control) × 2 (relational vs. domain) ANCOVA on relational retrieval, which showed a significant relationship with RPM as a covariate, *F* (1,76) = 6.57, *p* = .012, η^2^_p_ = .080. This analysis revealed a main effect of mindset wherein the participants who completed the analogy task retrieved more relational matches (*M* = 3.35, *SD* = 2.71) than those in the control conditions (*M* = 1.44, *SD* = 1.54), *F* (1,76) = 17.96, *p* < .001, η^2^_p_ = .191. Consistent with Jamrozik (2014), there was also a main effect of labels. Participants provided with relational labels at encoding retrieved significantly more relational matches (*M* = 3.67, *SD* = 2.42) than those provided with domain labels (*M* = 1.11, *SD* = 1.63), *F* (1,76) = 31.36, *p* < .001 η^2^_p_ = .191. Furthermore, these two factors interacted, *F* (1,76) = 7.84, *p* = .006, η^2^_p_ = .094. As can be seen in Fig. 3, the benefit of relational labels in eliciting more relational retrievals was greater for those in the mindset condition.

**Fig. 4:**
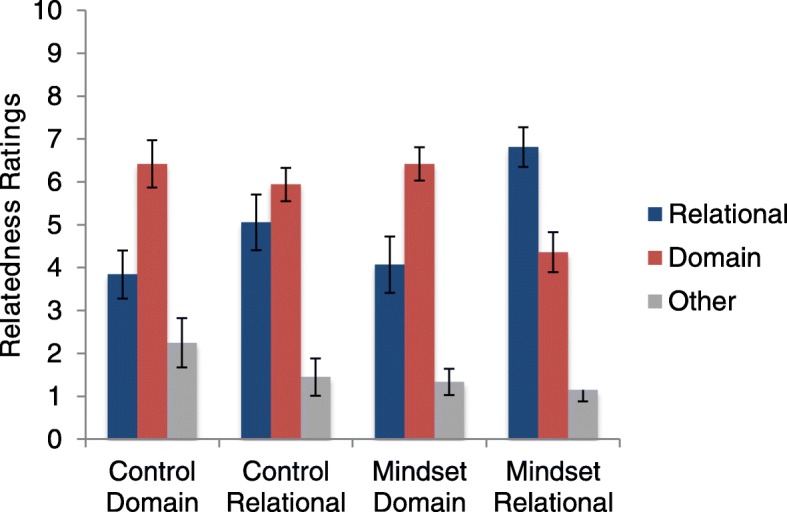
Means (and standard errors) of relatedness ratings in the text-mapping task in experiment 2. Original scale was 0–100

Next we conducted a 2 (mindset vs. control) × 2 (relational vs. domain) ANCOVA for the relational ratings in the text-mapping task, which did not show a significant relationship with RPM as a covariate, *F* (1,76) = 2.60, *p* = .11, η^2^_p_ = .033. A main effect of mindset revealed participants in the mindset condition rated relational matches as significantly more related (*M* = 5.68, *SD* = 2.92) than those in the control condition (*M* = 4.45, *SD* = 2.59), *F* (1,76) = 3.99, *p* = .049, η^2^_p_ = .050. Likewise, there was a main effect for labels, with the participants who were provided with relational labels rating relational matches higher (*M* = 6.11, *SD* = 2.66) than those who were provided with domain labels (*M* = 3.96, *SD* = 2.60), *F* (1,76) = 12.19, *p* = .001, η^2^_p_ = .138. The two factors did not show a statistically significant interaction, *F* (1,76) = 3.35, *p* = .07, η^2^_p_ = .042. Figure 4 shows the whole pattern.

The patterns across retrieval and text-mapping were quite similar. The relational mindset/relational labels condition elicited the most relational retrievals and the highest perceived similarity between relational matches. Does the boost from a relational mindset and relational labels for retrieval extend beyond the increased similarity? To answer this question, we re-conducted the above ANCOVA with relational retrieval as the dependent measure, but added the relational match text-mapping ratings as an additional covariate to RPM (showing the following relationships as covariates: RPM, *F* (1,75) = 3.94, *p* = .051, η^2^_p_ = .050; text-mapping ratings, *F* (1,75) = 24.08, *p* < .001, η^2^_p_ = .243). If the combination of inducing a relational mindset with relational labels only increased retrieval to the degree that it increased relational similarity, then the ANCOVA should *not* show significant effects on retrieval. However, this analysis showed that, yes, all three effects of retrieval (discussed above) replicate with the additional covariate of text-mapping rating. The main effects of relational mindset, *F* (1,75) = 13.12, *p* = .001, η^2^_p_ = .149, relational labels, *F* (1,75) = 16.90, *p* < .001, η^2^_p_ = .184, and their interaction, *F* (1,75) = 4.50, *p* = .037, η^2^_p_ = .057, were all statistically significant. Thus, although a relational mindset and relational labels increase perceived similarity between relational matches, their effect on retrieval goes beyond this increased perceived similarity.

Next, we analysed the picture-mapping task (this ANCOVA showed no significant relationship with RPM as a covariate, *F* (1,76) = 3.02, *p* = .086, η^2^_p_ = .038). Here we saw no main effect of mindset (mindset: *M* = 5.91, *SD* = 2.80; control: *M* = 5.36, *SD* = 2.57; *F* < 1), but we did see a main effect of relational labels, such that participants who were provided relational labels made more relational choices in the picture-mapping task (*M* = 6.33, *SD* = 2.58) than participants who were provided domain labels (*M* = 4.86, *SD* = 2.64), *F* (1,76) = 4.58, *p* = .036, η^2^_p_ = .057. In addition, there was a significant interaction between the two factors, *F* (1,76) = 8.66, *p* = .004, η^2^_p_ = .102, because relational labels elicited more relational choices than domain labels in the mindset condition (relational: *M* = 6.96, *SD* = 2.34; domain: *M* = 4.42, *SD* = 2.78), but did not in the control condition (relational: *M* = 5.39, *SD* = 2.70; domain: *M* = 5.59, *SD* = 2.32). A post-hoc test confirmed that the relational mindset/relational label condition elicited significantly more relational choices than any other condition, *F* (1,76) = 12.61, *p* = .001, η^2^_p_ = .142. This interaction suggests that relational labels can induce a relational mindset in a manner similar to how the analogy task did in prior research (Vendetti et al., 2014).

Finally, we analysed the relationships (correlations) among these key measures: relational retrieval, text-mapping ratings for the relational match passages, picture-mapping relational scores, and RPM scores (see Table 2). Again, we hypothesized that we would replicate the findings from Vendetti et al. (2014), who showed bigger correlations between RPM and the picture-mapping task for the control condition than for the mindset condition, and that this relationship would extend to the retrieval and text-mapping tasks. To be consistent with Vendetti et al. (2014) and experiment 1 we examined both correlations collapsing across conditions, and also for the mindset and control conditions independently (see Table 2). Unlike in experiment 1, where RPM showed strong relationships with the other three tasks collapsing across both mindset and control conditions, here RPM showed smaller relationships with the other three.

**Table 2:** Correlations (*r* scores) between relational retrieval, ratings of the relational match passage in the text-mapping task, relational choices in the picture-mapping task, and Raven’s Progressive Matrices (RPM) scores for experiment 2

Both conditions			*N* = 82
	Retrieval	Text-mapping	Picture-mapping
Retrieval			
Text-mapping	0.64*		
Picture-mapping	0.44*	0.38*	
RPM	0.05	0.03	0.09
Control condition			*n* = 36
	Retrieval	Text-mapping	Picture-mapping
Retrieval			
Text-mapping	0.48*		
Picture-mapping	0.15	0.08	
RPM	0.32*	0.40*	0.33*
Mindset condition			*n* = 46
	Retrieval	Text-mapping	Picture-mapping
Retrieval			
Text-mapping	0.68*		
Picture-mapping	0.56*	0.56*	
RPM	0.03	0.19	−0.06

*indicates p < .05

Separating the mindset and control conditions shows a different pattern from experiment 1, but a more similar to pattern to Vendetti et al. (2014), wherein the control condition showed bigger correlations between performance and RPM than the mindset condition. Here, the control condition shows numerically greater relationships between RPM and the other three tasks than the mindset condition (control: retrieval, *r* (34) = .322, text-mapping *r* (34) = .398, and picture-mapping, *r* (34) = .325; mindset: retrieval, *r* (44) = .027, text-mapping *r* (44) = −.191, and picture-mapping, *r* (34) = −.058) but only the difference in the size of the relationship with the text-mapping task was statistically significant across conditions, *z* = .266, *p* < .05.

A key part of the pattern from Vendetti et al. (2014) (experiment 1b in their paper, page 931) was that there was a significant relationship between RPM and picture-mapping scores in the control condition but not in the mindset condition (their results: control condition, *r* (26) = .41, *p* < .03; mindset condition, *r* (26) = .09, *p* > .64; their conditions combined: *r* (52) = .24, *p* = .08). Our findings from experiment 1 were not consistent with this pattern, but our findings from experiment 2 were consistent. Because of the importance of replication, we wanted to give the fairest assessment of the size of these relationships by including our entire sample across both experiments, see Table 3.

**Table 3:** Correlations (*r* scores) between relational retrieval, ratings of the relational match passage in the text-mapping task, relational choices in the picture-mapping task, and Raven’s Progressive Matrices (RPM) scores for experiments 1 and 2

Both conditions			*N* = 134
	Retrieval	Text-mapping	Picture-mapping
Retrieval			
Text-mapping	0.67*		
Picture-mapping	0.45*	0.42*	
RPM	0.21*	0.20*	0.23*
Control condition			*n* = 59
	Retrieval	Text-mapping	Picture-mapping
Retrieval			
Text-mapping	0.62*		
Picture-mapping	0.27*	0.34*	
RPM	0.35*	0.49*	0.36*
Mindset condition			*n* = 75
	Retrieval	Text-mapping	Picture-mapping
Retrieval			
Text-mapping	0.68*		
Picture-mapping	0.54*	0.48*	
RPM	0.14	−0.03	0.13

*indicates p < .05

Collapsing across experiments, we show rather similar sized relationships between RPM and the other measures as did Vendetti et al. (2014). In the control condition, the relationships between RPM and relational retrieval, *r* (57) = .349, text-mapping, *r* (57) = .492, and the picture-mapping score, *r* (57) = .364, were all statistically significant (*p*s < .05), while none were statistically significant for the mindset condition: relational retrieval, *r* (73) = .139; text-mapping, *r* (73) = −.027; and picture-mapping, *r* (73) = .127. We note, however, that one condition showing a significant effect and another difference not showing a significant effect does not mean that these two conditions are significantly different from each other. We report our results in this manner to follow how Vendetti et al., (2014) reported their results. In addition, we also directly contrasted the size of the correlations between the two conditions in order to evaluate the difference in their magnitude. The only difference of correlation size with RPM between conditions that was statistically significant was for text-mapping, *z* = 3.17, *p* < .01 (relational retrieval: *z* = 1.26, *p* = .21; picture-mapping: *z* = 1.42, *p* = .16).

To complete the comparison to Vendetti et al., who also reported total correlations without considering differences between conditions, we collapsed across conditions as well: retrieval, *r* (132) = .212; text-mapping, *r* (132) = .203; picture-mapping, *r* (132) = .225; all *p*s < .05.

In summary, although neither experiment 1 nor experiment 2 alone replicate the pattern from Vendetti et al. (2014) exactly, combining the results of experiments 1 and 2 show quite similar values to the prior research. This raises our confidence that the current experiments successfully tapped into the same cognitive processes as Vendetti et al. (2014), while highlighting the need to compare the magnitude of relationships across conditions without relying on a difference between a statistically significant versus nonsignificant finding.

## General discussion

We found evidence that inducing a relational mindset can increase analogical retrieval. However, we only found evidence for a relational mindset advantage when it is induced before encoding an initial set of passages to be retrieved later. Furthermore, we found evidence that this relational mindset advantage is due to an increased focus on, or higher quality encoding of, relational information in the initial passages, allowing these passages to be more readily cued by later relational matches. This explanation is supported by the interaction in experiment 2 between the use of relational labels and an induced relational mindset. The presence of the relational labels amplified the advantage of the relational mindset, suggesting that a relational mindset takes advantage of a clear, organized way to encode relational information.

However, we found no evidence that inducing a relational mindset at the time of retrieval increased analogical retrieval. It appears that if the relational information of the original examples were not encoded appropriately, attempting to induce a relational mindset does not make them any more accessible if they are needed later. This is consistent with the common computational explanations for the general rarity of relational retrieval (Forbus et al., 1995). Taken together, these results suggest that the key to relational retrieval is high-quality relational encodings, and offers two ways to improve encoding quality of learning materials. The first is using clear and prominent language that highlights the relevant relational concepts (e.g. using consistent relational labels for examples). The second is the use of analogical thinking exercises before engaging in study. Extending these findings to real-world learning contexts is the logical next step of this research.

We also need to consider potential limitations of these findings and alternative explanations for the results. One question is the source of the label × mindset interaction. We claimed that relational language amplified the positive effect of the mindset manipulation by providing a candidate relational structure to focus on in each example. Instead, could the domain label have acted as a suppressant, and is it possible to tell without having had a control condition without any passage labels? Our motivation for using the domain label was to offer each passage a “tag” in memory that might boost retrieval. There was no indication from prior work (Jamrozik, 2014) that providing domain labels would lessen relational retrieval.

Another concern with our interpretation of the label × mindset interaction (as pointed out by one of this manuscript’s reviewers) might be that participants did not know the relational labels used in the experiments, and so the relational labels may not have offered candidate relational structure in labelled examples. Previous work (Jamrozik, 2014) found that the relational labels were comprehensible when applied to examples, and those participants had seen most of the relational terms before. Our assumption was that the participants in the current study would likewise have enough familiarity with these terms that the label and example pairs would make the meaning clear enough. To be certain of this assumption, it would have been sensible to ask participants for their definitions of these terms at the end of the experiment. The reviewer who raised the issue suggested that perhaps nonce words could have had the same effect. This is an open empirical question, but previous work (Jamrozik, 2014) did not find nonce words to be comprehensible when paired with these passages, suggesting that they would not elicit a relational interpretation of examples.

A further issue that our experiments did not clearly resolve concerns how a relational mindset might be maintained or disrupted over time. Clearly, once induced, a relational mindset will not persist forever. Here, we can only offer speculation, but we hope this explanation spurs future research aimed directly at answering this question.

The condition that elicited the most relational retrievals was when participants performed the relational mindset task before encoding and read passages with clear relational labels. We believe that this combination increased the focus of the participants on relational information in the encoding passages that carried through to increase relational retrieval and further transferred to the picture-mapping task (which is of course the original evidence for a relational mindset), and then finally to the text-mapping task.

However, in experiment 2, when the relational mindset task was paired with passages with domain language, there was no effect of the relational mindset induction on the picture-mapping task. Likewise, in experiment 1 we did not find evidence that the relational mindset induction affected choices on the picture-mapping task. In both the relational mindset condition of experiment 1 and the relational mindset/domain labels condition of experiment 2, there were limited relational retrievals in between the mindset induction and the picture-mapping task. Perhaps once a relational mindset is induced, further success with relational processing is needed to maintain it. This continued success was achieved with the relational mindset/relational labels condition of experiment 2, which elicited high relational performance across a series of tasks. Likewise, in the original study by Vendetti et al. (2014), the picture-mapping task was completed immediately after the relational mindset analogy task and so there was no opportunity to disrupt the induced mindset. This explanation is post hoc, but the results fit the pattern that a relational mindset persists for as long as continued relational processing is successful. Consistent with this idea, the time between inducing a relational mindset and assessing its effects with retrieval and the picture-mapping task in the successful condition in experiment 2 was longer than when it failed to have an effect in experiment 1. This suggests that cognitive factors are important, and not simply time since induction.^3^

Overall, our findings were consistent with accounts of analogical retrieval and problem solving that emphasize the importance of high-quality relational encoding in maximizing the chances that our prior experiences are cued by future cases with common relational structure (e.g. Gentner et al., 2003). In this way, having a relational mindset when encoding a single case (with a relational label) had an effect similar to that observed previously due to comparing a pair of analogue cases. However, “the late abstraction principle” (Gentner et al., 2009) suggests that comparing two cases at the time of retrieval can also improve retrieval of prior cases with shared relational structure. We found no evidence that a relational mindset could elicit a similar effect from a single cue case at the time of retrieval. Likewise, we found no evidence that a relational mindset at the time of retrieval could elicit a relationally driven retrieval strategy. To be clear, this is not direct evidence against a strategic account of analogical retrieval (Dunbar & Blanchette, 2001), but it is a failure to find evidence for a plausible extension of this account.

We motivated this research by arguing for the importance of reliable ways to increase access to the right information in memory when it is needed at the time of problem solving. We tested whether inducing a relational mindset could join recent work by Minervino, Trench and colleagues that demonstrated methods to boost analogical retrieval at the time of problem solving. Trench et al. (2017) found that encouraging learners to create schematic perceptual representations of problems they were attempting to solve boosted analogical retrieval. Likewise, Minervino et al. (2017) found that encouraging learners to generate analogue problems of the problems they were attempting to solve boosted analogical retrieval. Unfortunately, inducing a relational mindset cannot be added to the available toolkit of ways to increase analogical retrieval while solving a problem. Given the potential utility of this exercise (solving four-word analogy problems), and the plausibility that it could have helped, it was worthwhile testing.

We end by noting not to be too pessimistic about these results. We found evidence that a relational mindset at the time of encoding was quite helpful. This suggests that solving some relatively easy four-word analogy problems is an effective way to “warm up your mind” in settings where you would want to prepare for future analogical retrieval, such as in engineering and design of education programs, or when reading source materials you hope to apply in the future. A clear next step should examine if this simple analogical thinking exercise boosts learning and transfer in these kinds of real-world educational and problem-solving settings.

## Supplementary information

**Additional file 1.** Encoding and retrieval stories.

**Additional file 2.** Analogy items (from Green et al., 2009).

**Additional file 3.** Picture-mapping images (from Markman & Gentner, 1993; Tohill & Holyoak, 2000; Vendetti et al., 2014).

## Data Availability

Materials are included in the manuscript and Additional file. Raw data are available at https://osf.io/qszv4/

## Footnotes

1 We note that Vendetii et al. (2014; experiment 1b) had *N* = 54 for their similarly designed experiment that showed the relational mindset advantage.

2 These sample sizes were arrived at by a combination of pragmatic restrictions and the results of Jamrozik (2014). That research found large effect sizes for the benefit of relational labels at encoding, *d* = 1.2, *N* = 60 for a four group between-participants experiment. Thus, we were confident we would be able to resolve key effects of interest with *N* > 80 for a four group between-participants experiment. We did not conduct analyses before sampling was complete.

3 We note it is unclear how the semantic word-association task completed during the delay between encoding and retrieval fits into this explanation.
