# Supplementary material for: Can a relational mindset boost analogical retrieval?
Source: Cogn Res Princ Implic. 2019 Dec 19;4:47. doi: 10.1186/s41235-019-0198-8 (PMC6923295; doi:10.1186/s41235-019-0198-8)
Supplement: Supplementary file 1 — Additional file 1. Encoding and retrieval stories. [file 41235_2019_198_MOESM1_ESM.docx]

**Additional file 1: Encoding and Retrieval Stories**

**Key Passages**

**Relational concept 1. *Energy transformation***

**Set A Domain: *Mechanical Engineering***

Relational-Label: This is an example of an *energy transformation*.

Domain-Label: This is an example from *mechanical engineering*.

Passage body: A car engine uses fuel, such as gasoline, to move the car. The way this fuel gets converted into motion is that pistons in the engine compress a mixture of fuel and air, which is ignited by the spark plugs. This mixture explodes, causing the pistons to move away in the opposite direction. The movement of the pistons powers the consecutive movement of car components that connect to the drive wheels, moving the car forward.

**Set B Domain: *Atmospheric science***

Relational-Label: This is an example of an *energy transformation*.

Domain-Label: This is an example from *atmospheric science*.

Passage Body: In certain areas of the world, such as in the Alps, the melting of ice can be used to produce electricity. When the glaciers found high in the mountains melt in the summer, water runs down the mountains and forms glacial rivers. The rivers can be diverted to pass through hydroelectric dams, where the force of the water rotates turbines connected to electrical generators. The rotation of the turbines causes the generators to produce electricity.

**Relational concept 2. *Common cause***

**Set A Domain: *Economics***

Relational-Label: This is an example of a *common cause*.

Domain-Label: This is an example from *economics.*

Passage Body: When a major factory shuts down, devastating changes can sweep through the region. For workers, a factory closing means loss of a job and, often, loss of the ability to pay for necessities like rent and food. For the town or city, a factory closing results in lower tax revenue. For the larger area, a factory closing tends to increase the costs of welfare and crime prevention.

**Set B: *Anatomy***

Relational-Label: This is an example of a *common cause*.

Domain-Label: This is an example from *anatomy.*

Passage Body: If the body experiences a sudden stressor, the sympathetic nervous system mobilises the body’s flight or flight response, resulting in rapid changes throughout the body. In the eyes, the pupils dilate to allow more light to enter. In the lungs, muscles relax to open the bronchioles and increase air flow. The response also impacts blood flow throughout the body: the heart beats faster and blood is directed away from the stomach and upper digestive system to slow digestion.

**Relational concept 3. *Differentiation***

**Domain Set A: *Anatomy***

Relational-Label: This is an example of *differentiation*.

Domain-Label: This is an example from *anatomy.*

Passage Body: Stem cells allow the body to efficiently replace cells that died or that were damaged. Stem cells can develop into many kinds of more specialised cells. For instance, one stem cell could develop into a long and skinny muscle cell that becomes part of the heart wall. Another stem cell could develop into a small and compact epithelial cell that becomes part of the digestive system lining.

**Set B: *Economics***

Relational-Label: This is an example of *differentiation*.

Domain-Label: This is an example from *economics.*

Passage Body: In some companies, hiring liberal arts graduates is a good way to fill gaps left when more experienced workers retire or find a new job. These new workers develop different specialised skills based on what job category they are assigned to. For example, someone working in accounting might develop skills related to managing databases. Someone working in human resources might develop skills related to communication.

**Relational Concept 4. *Positive feedback loop***

**Domain Set A: *Atmospheric science***

Relational-Label: This is an example of a *positive feedback loop*.

Domain-Label: This is an example from *atmospheric science.*

Passage Body: Global warming can result in escalating problems such as the melting of polar ice. Water absorbs more heat from sunlight than ice does. When polar ice is turned into water, this extra water retains additional heat. As a result, the temperature of the earth rises. This in turn leads to increased polar ice-melt, which then leads the earth’s temperature to rise even more rapidly.

**Domain Set B: *Electrical engineering***

Relational-Label: This is an example of a *positive feedback loop*.

Domain-Label: This is an example from *electrical engineering.*

Passage Body: Big problems can occur if a microphone is placed too close to a speaker. Any noise that the microphone picks up from the speaker gets amplified and played back through the speaker at a higher volume. When this louder noise is picked up by the microphone, it is reamplified and played back through the speaker at an even higher volume. The resulting noise is again reamplified and played back through the speaker, leading the noise to get increasingly louder.

**Relational Concept 5. *Self-regulation***

**Domain Set A: *Electrical engineering***

Relational-Label: This is an example of *self-regulation*.

Domain-Label: This is an example from *electrical engineering.*

Passage Body: The job of a power supply is to provide constant set voltage to the electronic device it feeds – such as a speaker. So, when the incoming voltage from the electric grid fluctuates upward, circuits inside the power supply operate to decrease the output voltage to the speaker. When the incoming voltage drops, these circuits act to increase the output voltage to the speaker.

**Domain Set B: *Mechanical engineering***

Relational-Label: This is an example of *self-regulation*.

Domain-Label: This is an example from *mechanical engineering.*

Passage Body: Steam locomotives had a device called the centrifugal governor, which was an early precursor to today’s cruise control. The governor controlled the speed of the locomotive’s engine by adjusting the rate of steam supplied to it. If the engine sped up above the preset speed, the governor would close off a valve to reduce the amount of steam going into the engine, slowing it down. Once the engine slowed, the valve would open again to allow more steam into it.

**Relational Concept 6. *Inoculation***

**Domain Set A: *Psychology***

 Relational-Label: This is an example of *inoculation*.

Domain-Label: This is an example from *psychology.*

Passage Body: A person’s attitudes can be changed by outside forces, such as persuasive advertisements. For example, after seeing a lot of convincing advertisements for cigarettes, a person’s attitude toward smoking might become more positive. In order to prevent persuasive arguments from changing a person’s attitude, the person can be asked to argue against weaker versions of these arguments. Later, if the person encounters more persuasive arguments, their attitude will not be affected.

**Domain Set B: *Medicine***

Relational-Label: This is an example of *inoculation*.

Domain-Label: This is an example from *medicine.*

Passage Body: People can become infected by viruses that they encounter in their environment, such as influenza or chicken pox. For instance, someone can become infected through contact with a contaminated doorknob or appliance. To prevent infection, people can be exposed to a less virulent form of a virus, which their bodies will fight off by developing antibodies against it. If they are then exposed to the full-strength virus, their bodies can effectively fight off an infection.

**Relational Concept 7. *Reciprocity***

**Domain Set A: *Political science***

Relational-Label: This is an example of *reciprocity*.

Domain-Label: This is an example from *political science.*

Passage Body: When countries enter a trade agreement, one country's governing body eliminates or limits fees for goods imported from the other country if they receive the same privilege for goods exported to that country. For example, the North American Free Trade Agreement allows free trade of certain items between North American countries. Countries that have trade agreements also tend to cooperate on other matters, such as work permits.

**Domain Set B: *Psychology***

Relational-Label: This is an example of *reciprocity*.

Domain-Label: This is an example from *psychology.*

Passage Body: In social interactions, one person’s positive action towards another is likely to lead the other person to be helpful and also respond with a positive action. For example, if one person helps another by doing an unrequested favor, the other person is likely to respond by later returning a favor. This pattern also extends to sharing information; people tend to share their knowledge with people who have helped them.

**Relational Concept 8. *Proportionality***

**Domain Set A: *Law***

Relational-Label: This is an example of *proportionality*. 
Domain-Label: This is an example from *law.*

Passage Body: The punishment for a crime should be reasonable given the seriousness of the crime. In the United States, this idea is detailed in the eighth amendment, which prohibits imposing excessive bail, fines, or cruel and unusual punishments. For example, in one landmark case, the Supreme Court ruled that a law that allowed someone to be fined over $300,000 for failing to report that he was taking money in excess of $10,000 out of the country was unconstitutional. Instead, a lower fine would have been more appropriate.

**Domain Set B: *Political science***

Relational-Label: This is an example of *proportionality*.

Domain-Label: This is an example from *political science.*

Passage Body: A governing body should only take as much action as is necessary to achieve its objectives. This principle was agreed upon by the European Union to limit the legislative and administrative powers of the governing body and to prevent it from becoming too involved in the affairs of its member countries. For example, if the European Union passes a treaty, it can only take as much action as is necessary to achieve the objectives of the treaty.

**Relational Concept 9. *Preemption***

**Domain Set A: *Computer science***

Relational-Label: This is an example of *preemption*.

Domain-Label: This is an example from *computer science.*

Passage Body: Certain tasks carried out by a computer system are more important than others. If a critical task needs to be carried out, the operating system can interrupt any of the less important tasks in order to complete the critical task. For instance, a computer system could be running security software that scans for threats that need to be blocked as soon as they are detected. If a threat is detected, the system can interrupt all of the less important tasks in order to block the threat.

**Domain Set B: *Law***

Relational-Label: This is an example of *preemption*.

Domain-Label: This is an example from *law.*

Passage Body: In the United States, federal law has priority over any particular state’s law. If a state law conflicts with federal law, the state law is invalidated and the federal law is followed. For example, one federal law specifies what information should be included on the labels of medical devices. When Massachusetts passed a law that required additional information on hearing aid labels, federal courts barred Massachusetts from enforcing this law.

**Relational Concept 10. *Trade-off***

**Domain Set A: *Medicine***

Relational-Label: This is an example of a *trade-off*.

Domain-Label: This is an example from *medicine.*

Passage Body: In the treatment of certain diseases, such as some localised cancers, the doctor and the patient have to make very tough choices. They can choose the most effective treatment that will eliminate the cancer but that is likely to have a number of uncomfortable negative side effects. Or, they can choose a less intensive treatment that cannot be guaranteed to fight off the cancer but that has fewer side effects.

**Domain Set B: *Computer science***

Relational-Label: This is an example of a *trade-off.*

Domain-Label: This is an example from *computer science.*

Passage Body: When deciding how to store large files, such as videos, computer users have to weigh their options. Users can store the files as they are, but the files will take up a lot of storage space. Alternatively, they can compress the files so that they take up less space, but then the files will take time to open because they’ll require a decompression algorithm.

**Filler Passages**

**1. *Acclimation***

**Domain Set A: *Biology***

Relational-Label: This is an example of *acclimation*.

Domain-Label: This is an example from *biology.*

Passage Body: Blubber is the thick layer of fatty tissue found under the skin of some animals, such as dolphins. Dolphins have a slightly thicker layer of blubber in the winter than in the summer. In the winter, the blubber helps insulate the dolphins against the cold water temperatures. When the ocean waters heat up in the summer, dolphins adapt by losing some of their blubber.

**Domain Set B: *Biology***

Relational-Label: This is an example of acclimation.

Domain-Label: This is an example from *biology.*

Passage Body: This is an example of acclimation. Grizzly bears grow thick, heavy fur coats in the winter in order to insulate their bodies against low temperatures. Once temperatures rise in the spring, the bears shed their winter coats. The bears’ summer fur coats are less dense and keep the bears from over-heating.

**Relational Concept 2. *Diffusion***

**Domain Set A: *Sociology***

 Relational-Label: This is an example of *diffusion*.

Domain-Label: This is an example from *sociology.*

Passage Body: The first home refrigerators became available to consumers in the late 1910s but were very expensive – a refrigerator cost more than a car. As a result, there were very few households with refrigerators. As technology improved and prices dropped, more and more people adopted the appliance. After World War II, refrigerators went into mass production and were adopted by nearly every household.

**Domain Set B: *Sociology***

Relational-Label: This is an example of *diffusion*.

Domain-Label: This is an example from *sociology.*

Passage Body: The microwave oven was invented after World War II based on technology developed during the war. The first commercially available microwaves were extremely large, heavy, and expensive. Technology improved in the 1960s and companies introduced smaller and cheaper home microwaves. The appliance was quickly adopted by consumers and by 1997, over 90% of American households owned a microwave.

**Relational concept 3. *Power projection***

**Domain Set A: *Military history***

 Relational-Label: This is an example of *power projection*.

Domain-Label: This is an example from *military history.*

Passage Body: The Falkland Islands are a territory of the United Kingdom located off the coast of Argentina. In 1982, Argentina invaded the islands, beginning the two-month long Falklands war. In response to the attack, the United Kingdom launched a large-scale naval assault to ward off the Argentine invasion. The naval fleet’s ability to fight and win the war reasserted the British Navy as an international power.

**Domain Set B: *Military history***

Relational-Label: This is an example of *power projection*.

Domain-Label: This is an example from *military history.*

Passage Body: In the Russo-Japanese war of 1904-1905, Japan and Russia fought over territorial and harbor rights of Manchuria and Korea. In the war-ending battle of Tsushima, Russia launched a naval attack that was met by the much-smaller Japanese fleet. Though it was smaller, the Japanese fleet destroyed nearly all of the Russian vessels during the battle. The Japanese victory raised Japan’s prestige in Europe and established its reputation as a naval power.

**Relational Concept 4. *Stability***

**Domain Set A: *Ecology***

Relational-Label: This is an example of *stability*.

Domain-Label: This is an example from *ecology.*

Passage Body: Coral reef ecosystems can be affected by large disturbances in the ocean, such as storms and tsunamis. If a reef is small and home to few plant and animal species, it may be unable to recover after a large storm. However, if a reef is large and is the home to many diverse species, it is more likely to recover.

**Domain Set B: *Ecology***

Relational-Label: This is an example of *stability*.

Domain-Label: This is an example from *ecology.*

Passage Body: The habitats of plants and animals living in tropical rain forests can be affected by fires. A large fire can have catastrophic effects on the ecosystems of smaller forests. The ecosystems of larger forests are more resilient, however, and the plant and animal species living there are more likely to make a recovery.
