# Supplementary material for: Can a relational mindset boost analogical retrieval?
Source: Cogn Res Princ Implic. 2019 Dec 19;4:47. doi: 10.1186/s41235-019-0198-8 (PMC6923295; doi:10.1186/s41235-019-0198-8)
Supplement: Supplementary file 2 — Additional file 2. Analogy items (from Green et al., 2009). [file 41235_2019_198_MOESM2_ESM.docx]

**Additional file 2: Analogy items** (From Green et al., 2009)

answer:riddle :: key:lock

 ash:fireplace :: lint:pocket

aspirin:pain :: muffler:noise

baker:cake :: scientist:discovery

basket:picnic :: holster:gun

basketball:hoop :: traveler:destination

blindness:sight, :: poverty:money

blizzard:snowflake :: army:soldier

bracelet:wrist :: moat:castle

burger:bun :: book:cover

cleanser:face :: absolution:sinner

eraser:pencil :: amnesia:memory

father:son :: inventor:invention

flock:goose :: constellation:star

foresight:future :: x-ray:bones

foundation:house :: premise:argument

furnace:coal :: stomach:food

hoof:hoofprint :: introduction:impression

immunisation:disease :: forewarning:surprise

jacket:zipper :: wound:suture

ketchup:tomato :: fuel:petroleum

kitten:cat :: spark:flame

knee:kneepad :: snail:shell

lambchop:lamb :: chapter:book

landscaper:lawn :: barber:hair

launchpad:helicopter :: divingboard:diver

lawschool:lawyer :: vineyard:wine

movie:screen :: lightning:sky

multiplication:product :: brewing:beer

nose:scent :: antenna:signal

orchard:apple :: neighbourhood:apartment

painting:canvas :: birthmark:skin

pen:pig :: reservoir:water

rectangle:perimeter :: nation:border

revising:manuscript :: evolving:species

saxophone:jazz :: typewriter:poetry

sugar:coffee :: incentive:deal
thermometer:temperature :: polygraph:honesty

train:track :: signal:wire

watermelon:rind :: cigarette:butt

**Semantic Word-Association Sets (#1)**

answer, riddle, guess, think

ash, fireplace, furnace, heat

 pain, aspirin, pain, relief

 baker, cake, oven, eggs

 picnic, basket, rug, park

 basketball, shot, player, hoop

 blindness, eyes, sight, glasses

snowflake, coat, chill, blizzard

 bracelet, clasp, wrist, gold

 burger, unhealthy, bun, fast

face, cleanser, rub, water

eraser, pencil, school, mistake

father, kindship, authority, son

goose, flock, honk, flap

predict, plans, future, foresight

foundation, house, scaffold, build

coal, ironmaking, heat, furnace

introduction, social, greeting, impression

disease, medicine, safe, immunisation

layers, jumper, zipper, close

ketchup, red, squeeze, tomato

kitten, cat, purr, tail

slime, shell, snail, slow

read, chapter, character, book

landscape, mow, lawn, dirt

launchpad, helicopter, wind, crash

degree, lawschool, effort, lawyer

movie, screen, popcorn, date

multiplication, product, maths, memorise

scent, perfume, nose, stink

apple, worm, harvest, orchard

canvas, painting, interpretation, artist

pen, pet, pig, pink

perimeter, measurement, geometry, rectangle

revising, write, manuscript, draft

saxophone, jazz, performance, music

coffee, sugar, mug, morning

temperature, mercury, measure, thermometer

track, carriage, train, transport

watermelon, flesh, seeds, rind

**Semantic Word-Association Sets (#2)**

key, lock, door, fit

lint, pocket, clothing, fluff

muffler, noise, backfire, explosion s

cientist, discovery, experiment, hypothesis

holster, gun, cowboy, belt

traveler, destination, map, transport

poverty, poor, disease, suffer

army, soldier, wounds, war

moat, castle, drawbridge, secure

book, cover, read, writing

absolution, sinner, confess, guilt

loss, amnesia, memory, mind

inventor, new, innovation, create

constellation, star, galaxy, space

 x-ray, bone, radiation, machine

 premise, argument, convince, language

stomach, food, digest, enzyme

 hoof, hoofprint, horse, track

 forewarning, surprise, shock, prepare
 wound, suture, medic, heal

petroleum, fuel, energy, car

spark, fire, heat, burn

knee, kneepad, protect, shield

lambchop, lamb, carnivore, cook

hair, chop, style, barber

divingboard, diver, height, pool

vineyard, wine, agriculture, produce

lightning, sky, weather, thunder

brewing, beer, yeast, alcohol

antenna, signal, reception, wire

neighbourhood, apartment, block, house

birthmark, skin, pigment, unique

reservoir, water, ecosystem, land

nation, border, territory, country

evolving, adapt, genes, species

typewater, poetry, writer, tradition

incentive, deal, agreement, persuade

polygraph, honesty, liar, truth

signal, wire, electric, current

cigarette, butt, tobacco, filter
