# Supplementary figures and images for: Can a relational mindset boost analogical retrieval?
Source: Cogn Res Princ Implic. 2019 Dec 19;4:47. doi: 10.1186/s41235-019-0198-8 (PMC6923295; doi:10.1186/s41235-019-0198-8)

**Additional file 3: Picture-Mapping images** (from Vendetti et al., 2014; Tohill & Holyoak, 2000; Markman & Gentner, 1993)

1.


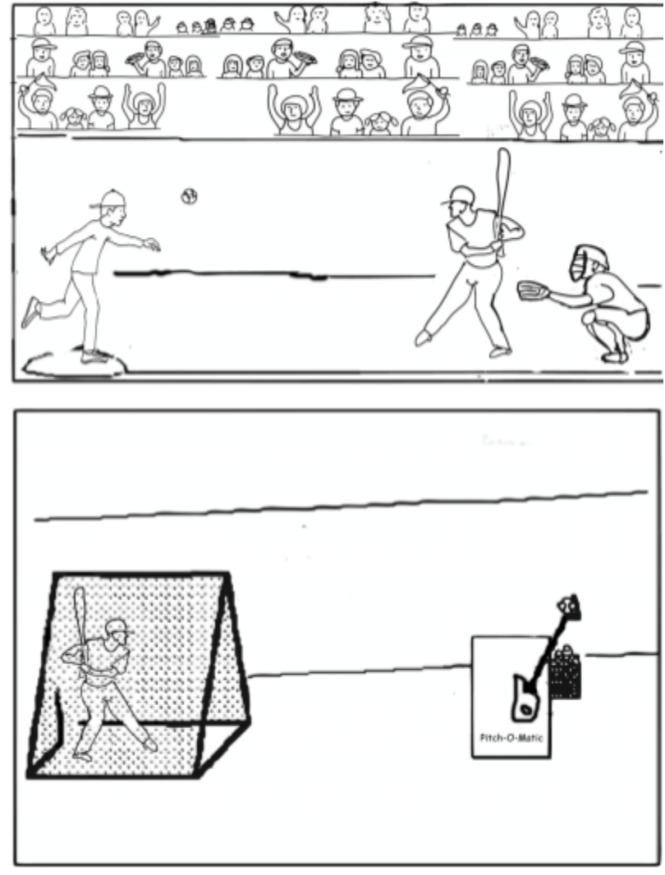


2.


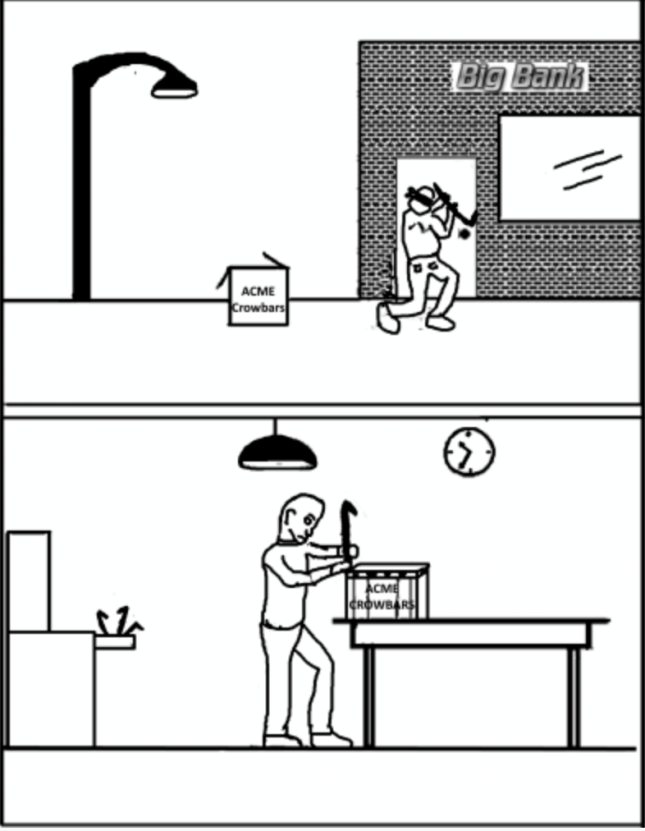


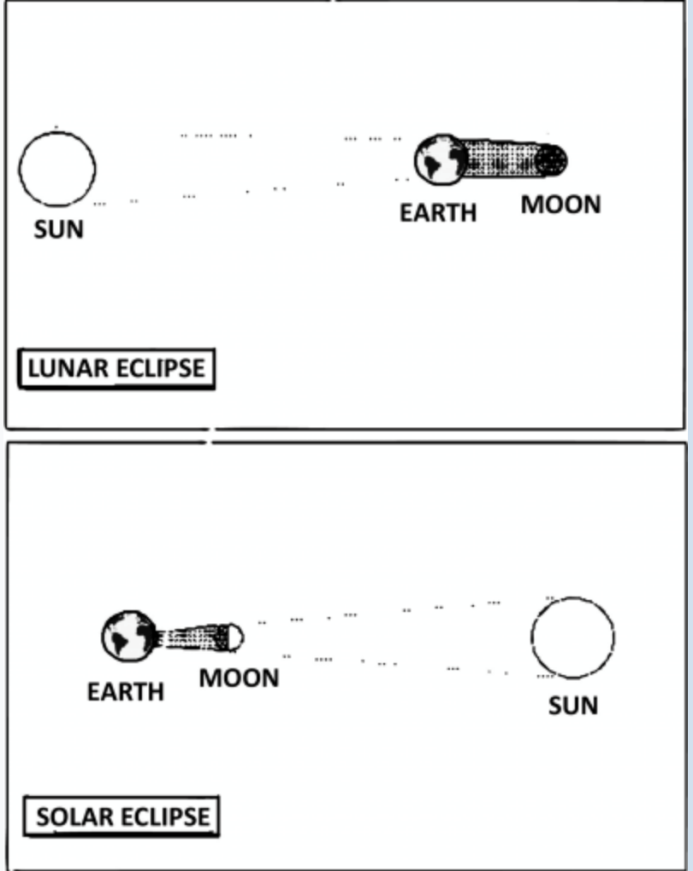


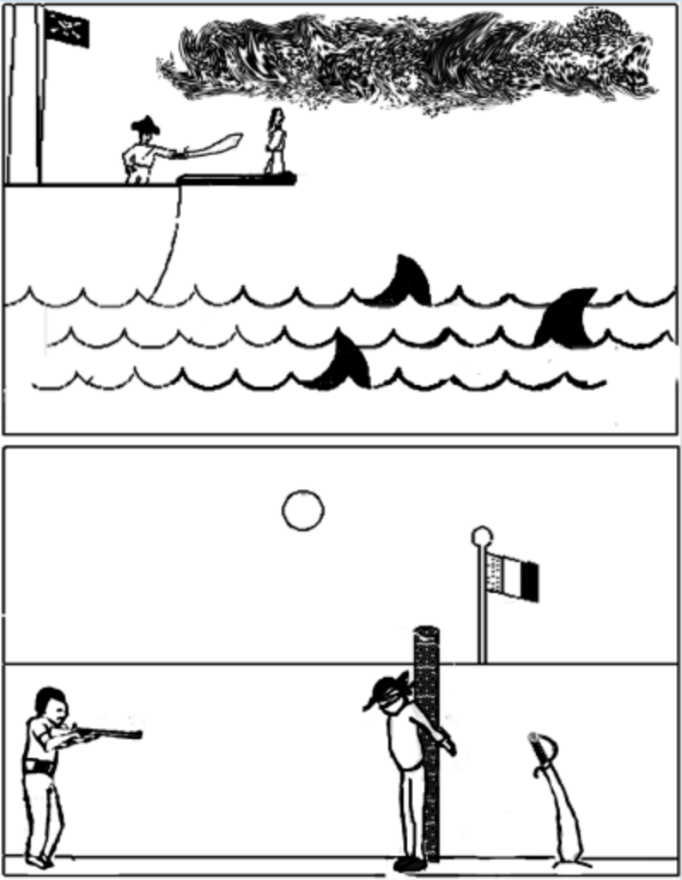


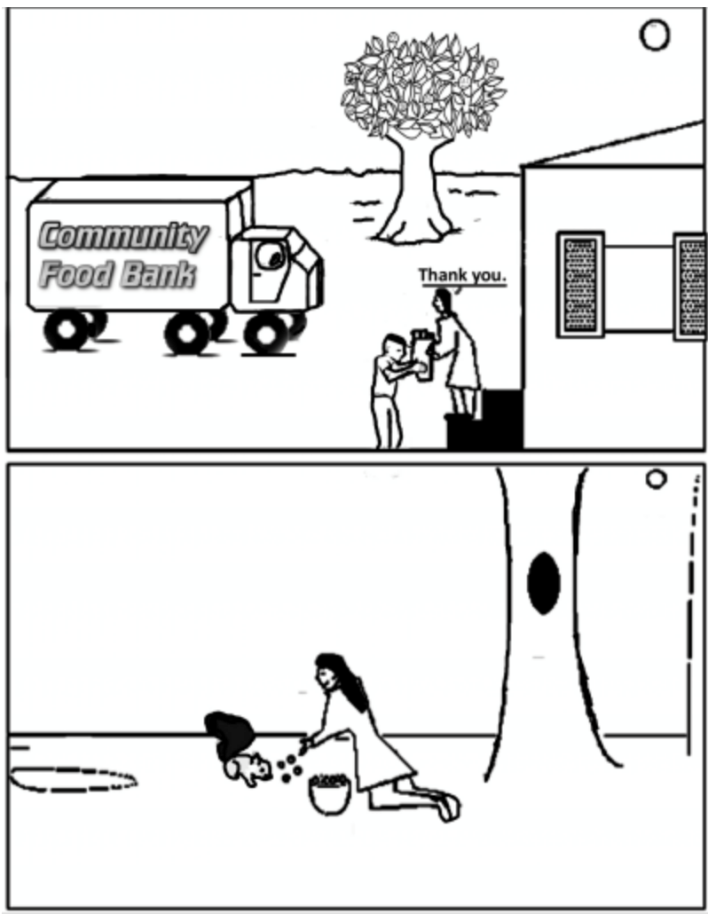


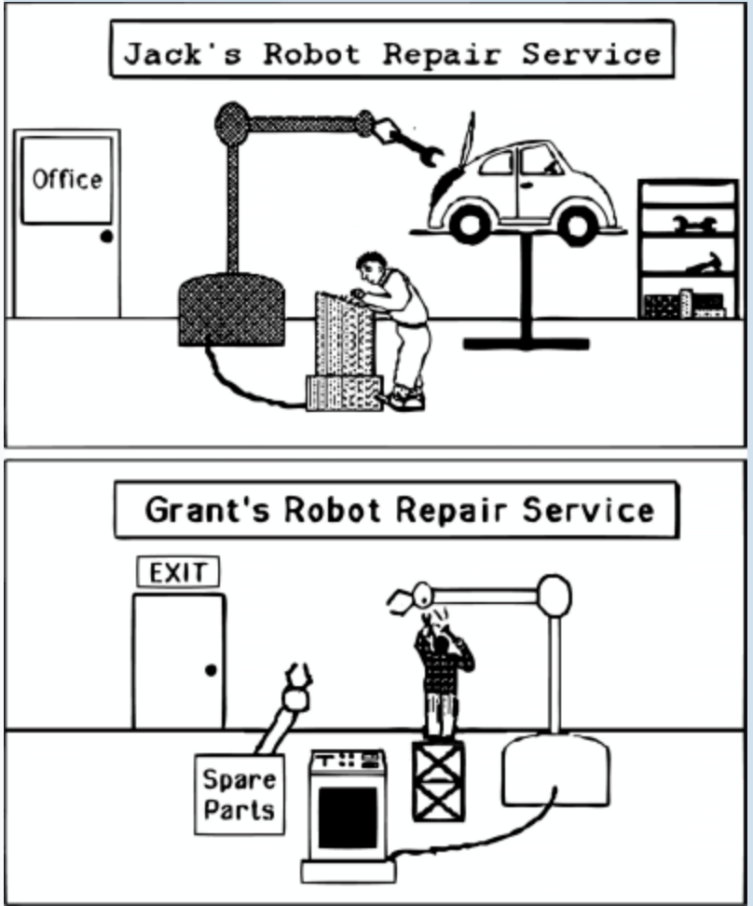


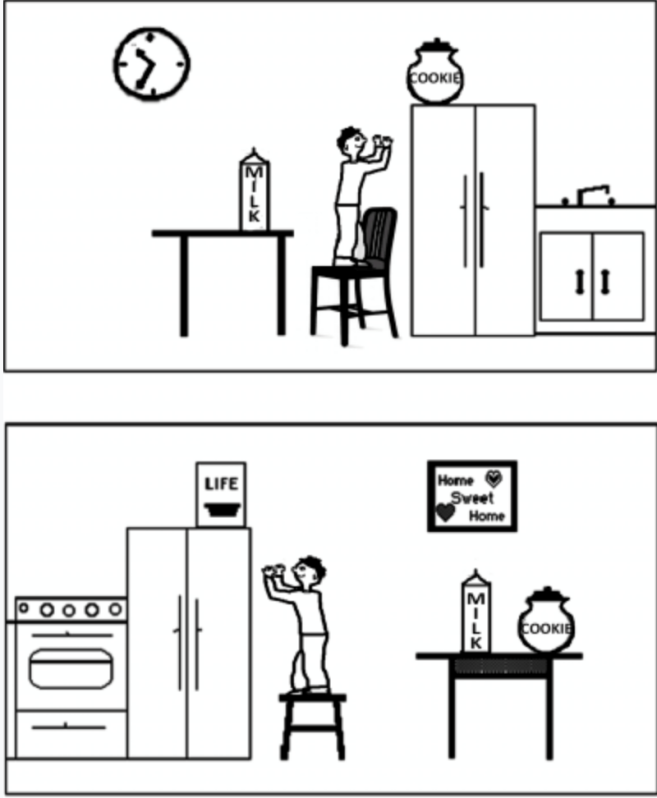


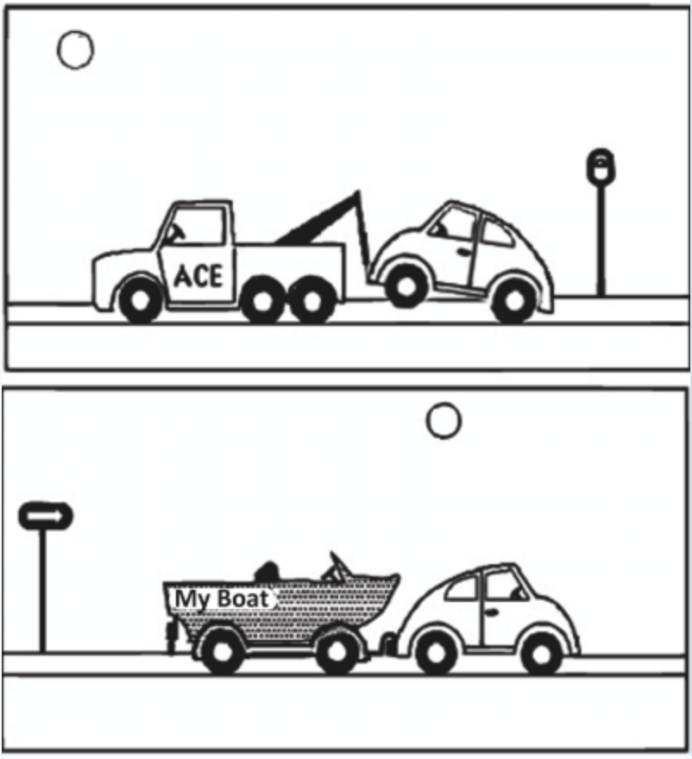


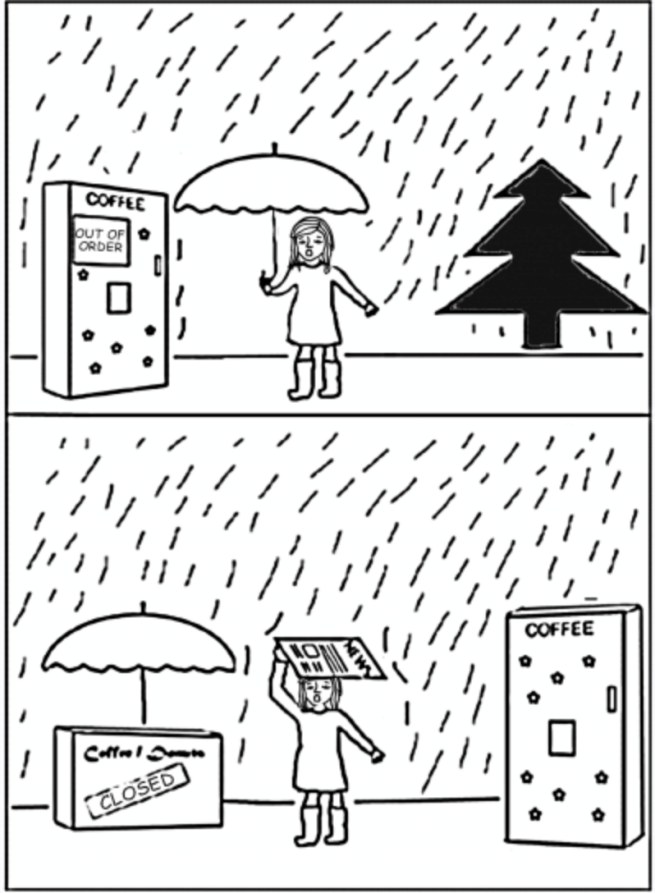


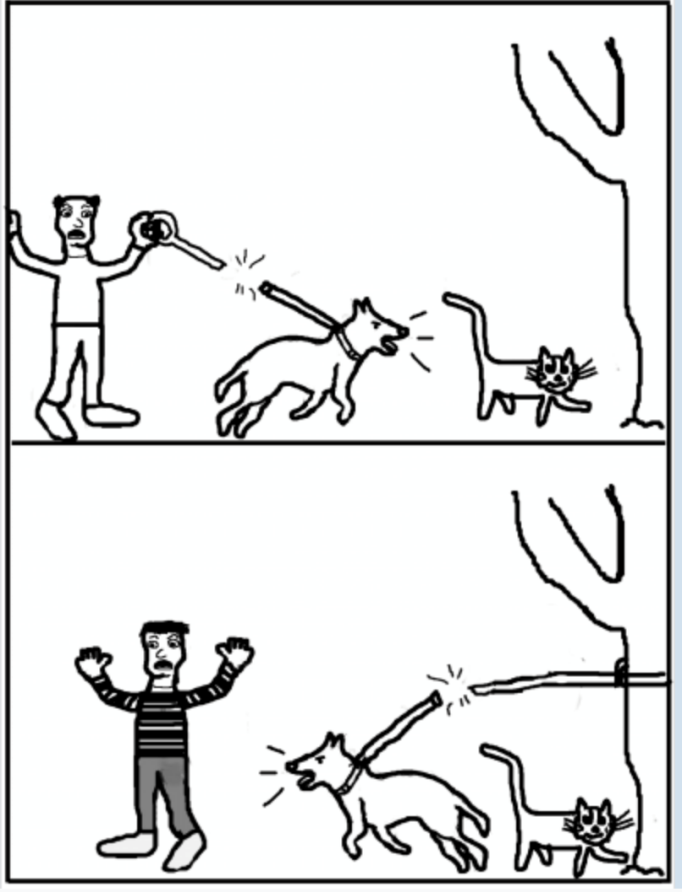

Supplement: Supplementary file 3 — Additional file 3. Picture-mapping images (from Markman & Gentner, 1993; Tohill & Holyoak, 2000; Vendetti et al., 2014). [file 41235_2019_198_MOESM3_ESM.docx]
